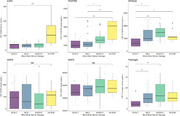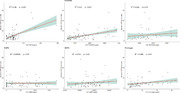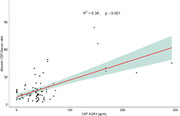# CSF profiling of impaired Blood‐brain barrier individuals: novel putative biomarkers discovery

**DOI:** 10.1002/alz.092219

**Published:** 2025-01-09

**Authors:** Giovanni De Santis, Kaj Blennow, Henrik Zetterberg, Nicholas J. Ashton

**Affiliations:** ^1^ Department of Psychiatry and Neurochemistry, Institute of Neuroscience and Physiology, The Sahlgrenska Academy, University of Gothenburg, Mölndal, Gothenburg Sweden; ^2^ Clinical Neurochemistry Laboratory, Sahlgrenska University Hospital, Mölndal Sweden; ^3^ Department of Psychiatry and Neurochemistry, Institute of Neuroscience and Physiology, The Sahlgrenska Academy, University of Gothenburg, Mölndal Sweden; ^4^ Wisconsin Alzheimer's Disease Research Center, University of Wisconsin School of Medicine and Public Health, Madison, WI USA; ^5^ Dementia Research Centre, Department of Neurodegenerative Disease, UCL Queen Square Institute of Neurology, University College London, London, United Kingdom, London United Kingdom; ^6^ Hong Kong Center for Neurodegenerative Diseases, Clear Water Bay Hong Kong; ^7^ UK Dementia Research Institute at UCL, London United Kingdom; ^8^ Department of Old Age Psychiatry, Institute of Psychiatry, Psychology, and Neuroscience, King’s College London, London, London United Kingdom; ^9^ King’s College London, Institute of Psychiatry, Psychology & Neuroscience, Maurice Wohl Clinical Neuroscience Institute, London United Kingdom

## Abstract

**Background:**

Blood‐brain barrier (BBB) integrity is crucial for brain homeostasis and maintenance. This is a pilot study to investigate cerebrospinal fluid (CSF) levels of several proteins implicated in BBB integrity, such as aquaporin‐4 (AQP4), platelet‐derived growth factor (PDGFRβ), human major facilitator superfamily domain containing protein 2A (MFSD2A), matrix metalloproteinase (MMP)‐9, Matrix metalloproteinase (MMP)‐2, and Fibrinogen, for assessing BBB integrity.

**Method:**

CSF samples were collected from 100 participants (36 [36%] female and 64 males [64%]; mean [SD] age, 73,34 [9,05] years). Participants were stratified according to their CSF/serum albumin concentration ratio (Q‐Alb); intact BBB (Q‐Alb <9, n = 25), mild BBB damage (Q‐Alb 9‐14, n = 25), modest BBB damage (Q‐Alb 14‐30, n = 24), and severe BBB damage (Q‐Alb >30, n = 25). AD CSF biomarkers (p‐Tau181, total‐Tau, Aβ42/40 ratio) were assessed by LUMIPULSE G1200. Protein levels were quantified using ELISA Kits (CUSABIO, Thermofisher, Bioss Antibodies). Correlation analyses were conducted between protein levels and albumin CSF/serum ratios with R (Kruskal‐Wallis test with pairwise comparison, Bonferroni method, was performed).

**Result:**

CSF AQP4 and PDGFRβ levels were significantly higher in individuals with severe BBB damage (Figure 1). No significant differences are highlighted in the levels of MMP9, MMP2, MFSD2A and Fibrinogen; whereas, in both MFSD2A and Fibrinogen it is shown a notable difference in the levels of protein between the modest and intact groups, with a subsequent decline in the severe group. A positive correlation (Figure 2) between PDGFRB and AQP4 with Q‐Alb (PDGFRB, r = 0,273, P < 0,0001; AQP4, r = 038, P < 0,001) was found, where AQP4 seems to best reflect albumin stratification (Figure 3).

**Conclusion:**

In this pilot study, we show that CSF concentration of some blood‐brain barrier‐related proteins increases with severity of BBB damage. This highlights their potential as novel biomarkers for monitoring BBB integrity. Remarkably, the presence of high concentrations of Fibrinogen in the CSF could be an important feature to further investigate.